# Health‐related quality of life outcomes after surgical treatment of atypical femur fractures: a multicenter retrospective cohort study

**DOI:** 10.1002/jbm4.10514

**Published:** 2021-09-30

**Authors:** Jonathon Spanyer, Lauren A. Barber, Harrison Lands, Alexander Brown, Mary Bouxsein, Marilyn Heng, Madhusudhan Yakkanti

**Affiliations:** ^1^ OrthoCincy Orthopaedic Surgery Edgewood Kentucky USA; ^2^ Hospital for Special Surgery Orthopaedic Surgery New York New York USA; ^3^ Dartmouth‐Hitchcock Health System Lebanon New Hampshire USA; ^4^ Andrews Sports Medicine and Orthopaedic Center Birmingham Alabama USA; ^5^ Center for Advanced Orthopedic Studies, Beth Israel Deaconess Medical Center Boston Massachusetts USA; ^6^ Orthopaedic Trauma Surgery Massachusetts General Hospital Boston Massachusetts USA; ^7^ Louisville Orthopaedic Clinic Louisville Kentucky USA

**Keywords:** ATYPICAL FEMUR FRACTURE, BISPHOSPHONATES, HRQOL, OSTEOPOROSIS, QUALITY OF LIFE, SF‐36

## Abstract

The objective of this study was to examine the health‐related quality of life (HRQOL) outcomes for surgically‐treated atypical femur fractures (AFFs) compared to typical femoral diaphyseal fractures. Two large trauma center databases were retrospectively queried for surgically‐treated femur fractures. Fractures were grouped into AFFs and compared to a control cohort. Controls for the AFF group included women with diaphyseal fractures without additional AFF characteristics. Patients were contacted for administration of the Short Form‐36v2 Health Survey. Surveys were completed an average of 30.3 months (range, 6–138 months) and 25.5 months (range, 5–77 months) postoperatively for the AFF and non‐AFF groups, respectively. All patients were female, with 46 patients in the AFF and 26 patients in the non‐AFF group. The average age of the AFF group was 70.1 years compared with an average age of 67.4 years in the non‐AFF group (*p* = 0.287). Over 90% (91.3%) of patients in the AFF group had a history of bisphosphonate use while 26.9% of patients in the non‐AFF group had used bisphosphonates (*p* < 0.0001). Patients with AFF reported their postoperative physical and mental health to be no different than similarly aged patients with femoral diaphyseal fractures, as measured by the Short Form 36, version 2 (SF‐36v2) Health Survey. These data suggest that mid‐term patient‐reported quality of life outcomes are similar among women who sustain an AFF compared to a cohort of more typical femoral diaphyseal fractures. © 2021 The Authors. *JBMR Plus* published by Wiley Periodicals LLC. on behalf of American Society for Bone and Mineral Research.

## Introduction

Bisphosphonates are currently one of the most commonly prescribed medications to prevent osteoporotic fractures.^(^ ^1^, ^2^, ^3^ ^)^ Bisphosphonates are frequently used as first‐line agents for postmenopausal osteoporosis.^(^ ^4^ ^)^ Alendronate was first synthesized in the 1970s, and by 2006 in the Unites States about 30 million prescriptions were written annually, accounting for nearly 15% of postmenopausal women.^(^ ^5^ ^)^ Because the incidence of osteoporotic fractures is expected to increase with the aging population, the prescriptions of bisphosphonates had also been predicted to increase.^(^ ^6^ ^)^ Yet with the advent of newer medications to treat osteoporosis, and with concerns about rare side effects from bisphosphonates such as osteonecrosis of the jaw and atypical femoral fractures (AFFs), a relative decrease in projected bisphosphonate utilization in the past decade has been realized.^(^ ^7^, ^8^, ^9^ ^)^

**Fig. 1:**
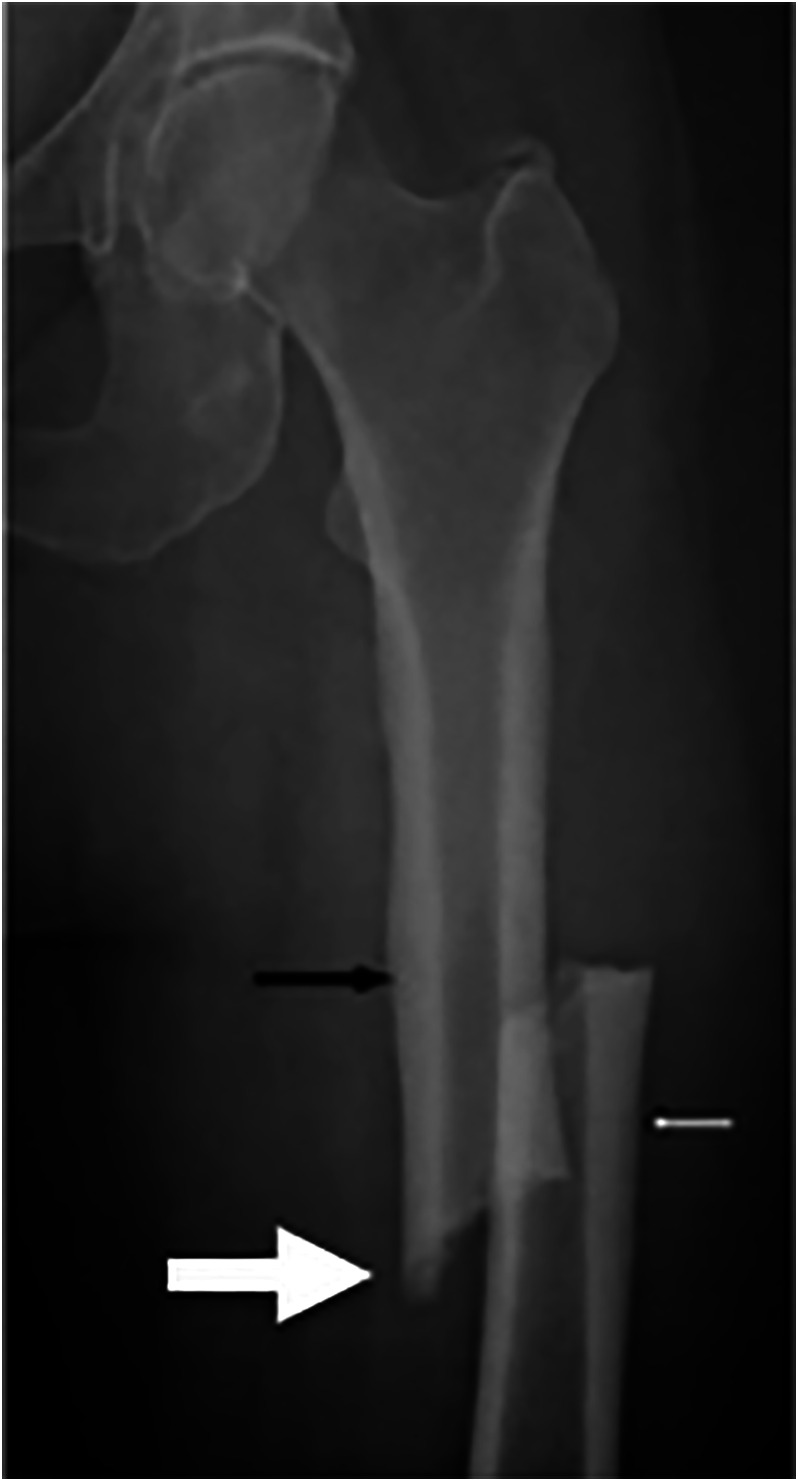
Representative atypical femur fracture radiograph. A 65‐year‐old female patient was taking bisphosphonates for 6 years, with 6 months of progressive prodromal thigh pain. She had seen an orthopedic surgeon 1 month prior to the fracture with pelvic x‐rays showing no evidence of significant osteoarthritis at the time. Note the periosteal thickening of the lateral cortices (black and white arrows), noncomminuted transverse fracture, and the medial cortical spike (big arrow) that are typically seen in atypical femur fractures.^(^ ^26^ ^)^

Not limited to management of osteoporosis, bisphosphonates have been used to treat a variety of pathologies ranging from Paget's disease of bone to hypercalcemia of malignancy. Safety profiles for bisphosphonates show they are generally well‐tolerated, but more recently there have been concerns about the long‐term use of bisphosphonates.^(^ ^1^, ^10^, ^11^ ^)^ In 2005, Odvina et al.^(^ ^12^ ^)^ first reported on a small number of patients who experienced primarily non‐spinal fractures of the lower extremity and femur while on bisphosphonate therapy, with histological analysis showing markedly suppressed bone formation after prolonged bisphosphonate use. Subsequently, several authors have reported similar findings, all associated with chronic bisphosphonate use.^(^ ^6^, ^13^, ^14^ ^)^ In each case, the fractures were found to be secondary to low‐energy mechanisms, presented with prodromal thigh pain, and demonstrating a prominent femoral cortex medial spike radiographically (Figure 1).^(^ ^15^, ^16^, ^17^ ^)^ Bilateral AFFs have been reported in up to 48% of cases.^(^ ^18^, ^19^, ^20^, ^21^, ^22^ ^)^ Cortical thickening near the fracture site and delayed union after surgical repair have also been reported.^(^ ^17^, ^23^, ^24^, ^25^ ^)^

A taskforce committee report commissioned by the American Society for Bone and Mineral Research (ASBMR) has established definitions of the emerging phenomenon to standardize investigations into the pathophysiology, epidemiology, and orthopedic clinical and medical management of AFFs.^(^ ^26^ ^)^ Numerous authors have reported on patients who have sustained AFFs, including presentations, treatments, duration and type of bisphosphonate use, and final clinical outcomes.^(^ ^27^, ^28^ ^)^ Although the radiographic characteristics and potential risk factors for AFFs have been well‐described, less has been reported about the health‐related quality of life (HRQOL) outcomes after patients have undergone surgical treatment for their AFF.

The objective of this study was to collect HRQOL outcomes from patients who underwent surgical treatment for their AFFs and compare them to a similar cohort who underwent surgical treatment for diaphyseal femoral fractures. In this way, we aimed to determine the relative effect of repaired AFFs compared with an otherwise healthy cohort sustaining a similar isolated fracture on quality of life measures.

## Patients and Methods

**Fig. 2:**
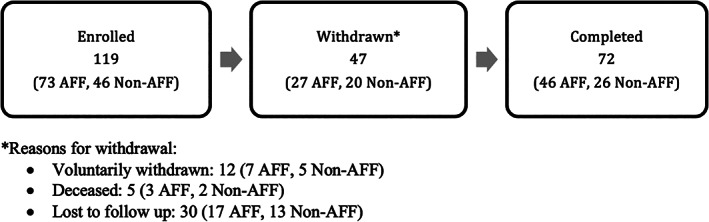
Cohort flowchart. Flowchart diagrams for the combined cohorts detailing enrolled participants and those withdrawn for various reasons to make up the completed participants. Abbreviation: AFF, atypical femur fracture.

After Institutional Review Board (IRB) approval, two large trauma center institution databases were queried for all surgically treated fractures of the femur using International Classification of Diseases, Ninth Revision (ICD‐9) codes from January 2004 to December 2014 at the Massachusetts General Hospital (MGH) and the University of Louisville. Only female patients were enrolled (Figure 2). Fractures of the hip and supracondylar regions were excluded from the study, leaving diaphyseal femoral fractures for review. All potential patient radiographs were reviewed by two orthopedic surgeons who were blinded to clinical details, and only fractures without previous implants or injuries were included. Based on imaging characteristics and mechanisms of injury, fractures were classified as either AFFs or non‐AFFs according to the 2014 ASBMR guidelines.^(^ ^26^ ^)^ Bisphosphonate use and duration was not used to determine fracture type.

Medical records were reviewed for use of any anti‐osteoporosis medication, with the focus on bisphosphonates, in the time preceding the fracture. Patients from both groups were then contacted via telephone and/or direct mailings where the Short Form 36, version‐2 (SF‐36v2) Health Survey and a brief survey of injury mechanism, medication use, smoking, employment, and previous fracture history was administered. Mean Physical Component Score (PCS) and Mental Component Score (MCS) were calculated according to the methods described by Taft et al.^(^ ^29^ ^)^ The average duration of bisphosphonate use was calculated. If a range of bisphosphonate use duration was given for a patient, the average number was used and those who had used bisphosphonates only once, were assigned a duration of 1 year because the majority of bisphosphonate medications have a relatively long half‐life. Additionally, mechanisms of injury were categorized as either high energy or low energy. Statistical analysis was performed using Microsoft Excel (Microsoft Corp., Redmond, WA, USA) for Student *t* tests and the MedCalc online statistical calculator (MedCalc Software Ltd., Ostend, Belgium; https://www.medcalc.org/) for proportion tests.

## Results

A total of 119 patients were identified with diaphyseal femoral fractures, and 72 patients (61%) were available for follow‐up; all patients completed the SF‐36v2 and brief health survey. Of these, 46 AFFs and 26 controls with isolated diaphyseal femur fractures were identified using the major and minor criteria outlined by the ASBMR (Table 1).^(^ ^26^ ^)^

**Table 1:** Major and minor criteria defining atypical femur fractures by the American Society for Bone and Mineral Research, taken from Shane et al.^(^ ^26^ ^)^

Parameter	Major features	Minor features
Fracture history	No or minimal trauma	Prodromal pain in the groin or thigh History use of other pharmacologic agents such as glucocorticoids or bisphosphonates
Location	Subtrochanteric or femoral shaft	Bilateral
Configuration	Transverse or short oblique Noncomminuted Medial cortical spike	Localized periosteal reaction of the lateral cortex Generalized cortical thickening Signs of delayed healing

The AFF and non‐AFF groups had similar ages: 70.1 ± 8.8 years (mean ± standard deviation) for the AFF group and 67.4 ± 10.8 years for the control group (*p* = 0.287) (Table 2). Over 90% (91.3%) of patients in the AFF group had used bisphosphonates, whereas 26.9% of patients in the non‐AFF group had used bisphosphonates (*p* < 0.0001). Average follow‐up between the two groups was similar at 30.3 ± 29.1 and 25.5 ± 18.7 months for the AFF and control groups, respectively (*p* = 0.397). The average duration of bisphosphonate use was 9.4 ± 5.6 years in the AFF group and 5.6 ± 4.0 years in the non‐AFF group (*p* = 0.060). The mechanisms of injury differed significantly between the two groups, with the AFF group generally experiencing lower energy mechanisms (ground‐level falls and fractures while simply walking) as compared to the higher‐energy mechanisms (motor vehicle accidents) in the control group. Lower‐energy mechanisms accounted for 95.7% of the fracture mechanisms in the AFF group versus 69.2% in the non‐AFF group (*p* = 0.002).

**Table 2:** Group comparisons

Group variable	AFF	Non‐AFF	*p*
Patients, *n*	46	26	
Age at fracture (years), mean ± SD	70.1 ± 8.8	67.4 ± 10.8	0.287
Bisphosphonate use, *n* (%)	42 (91.3)	7 (26.9)	**<0.0001**
Bisphosphonate duration (years), mean ± SD	9.4 ± 5.6	5.6 ± 4.0	0.060
Time postoperation to survey follow‐up (months), mean ± SD	30.3 ± 29.1	25.5 ± 18.7	0.397
Number low energy mechanism, *n* (%)	44 (95.7)	18 (65.4)	**0.002**
PCS score, mean ± SD	38.5 ± 10.5	35.9 ± 10.4	0.323
MCS score, mean ± SD	52.7 ± 11.5	51.4 ± 11.7	0.636

*Notes*: Demographic comparison between the atypical femur fractures group and age‐matched controls with similar fractures. All composite scores were based on normative values from the SF‐36v2 Health Survey. Bold values are significant.

Abbreviations: AFF, atypical femur fracture; MCS, mental component score; PCS, physical component score.

Patients with AFFs rated mid‐term postoperative physical and mental health similar to that of non‐AFFs, as measured by the PCS at 38.5 ± 10.5 for AFFs versus 35.9 ± 10.4 for non‐AFFs (*p* = 0.323) and the MCS at 52.7 ± 11.5 for AFFs versus 51.4 ± 11.7 for non‐AFFs (*p* = 0.636) calculated from the SF‐36v2.

## Discussion

In this study, we aimed to assess self‐reported health‐related quality of life (HRQOL) outcomes in women who sustained atypical femur fractures compared to those with isolated femoral diaphyseal fractures, both of whom underwent surgical repair of their fractures. The SF‐36v2 questionnaire used to assess an individual's health status, which can be used to compare outcomes between groups of patients by type of intervention or disease. The questionnaire consists of eight scales yielding two summary measures: PCS and MCS. With an average follow‐up time of 2.4 years, both groups reported similar postoperative PCS and MCS.

Our finding of similar self‐reported HRQOL between AFF and non AFF patients was unexpected, given that the AFF group generally experienced lower‐energy trauma (ground level fall, break while walking), compared to the non‐AFF group. Other authors have shown that high‐energy trauma can adversely affect HRQOL outcomes in orthopedic patients.^(^ ^30^ ^)^ However, Ko and Chang^(^ ^31^ ^)^ also reported on long‐bone fractures with similar outcomes for overall SF‐36v2 scores between higher‐energy femoral shaft fractures and lower‐energy isolated tibial shaft fractures after intramedullary nailing and subsequent implant removal and healing. Yet when divided into the survey's domains, the PCS had a higher score for femoral shaft fracture patients (*p* = 0.002).^(^ ^31^ ^)^ Our study suggested that although generally lower‐energy mechanisms were involved in the AFF group, the health effect upon the patient was still similar to the higher‐energy traumas in the non‐AFF group. The clinical significance of our study is that patients with AFF may expect similar mid‐term patient‐reported physical and mental outcomes to their higher‐energy non‐AFF counterparts.

Although our study suggests similar mid‐term outcomes between the cohorts after healing, other authors have reported higher rates of early postoperative complications among AFF patients with bisphosphonate use. A study by Edwards et al.^(^^)^ in 2013 reviewed data from the United States Food and Drug Administration Adverse Event Reporting System (FAERS), which revealed that 26% of cases of AFFs exhibited delayed healing or non‐healing. Additionally, Bogdan et al.^(^^)^ in 2015, reported a 12% failure rate as well as delayed average time to union after surgical repair of AFFs.

Although we realize that bisphosphonates remain a choice of therapy for individuals at high risk for fracture, the potential concern of AFFs has come to the attention of practitioners and patients, and prescriptions for bisphosphonates have declined in the past decade. However, the fracture prevention benefits of bisphosphonates remain, and it should be noted that the overall risk–benefit profile of bisphosphonates should be carefully considered in at‐risk patient populations. The current study indicates that mid‐term health‐related outcomes following surgical repair of AFF are similar to those of surgically‐repaired diaphyseal fractures of the femur.

Our study had several limitations, including the retrospective nature of data collection in this cohort study with limited sample size. Only those subjects who survived after fracture repair were available to enroll in the study, thereby possibly introducing a survival bias. A large number of patients were also lost to follow‐up in this cohort, suggesting that our results may not be generalizable to the population at large. Still, to our knowledge, this represents one of the largest cohorts of AFFs studied, and further provides HRQOLs measures for these AFF patients.

In spite of the small number of patients, to our knowledge, this is the first study to include standardized HRQOL outcomes, SF‐36v2, for patients treated surgically for AFFs. The information presented will be useful to the practicing orthopedic surgeon and the medical community, particularly with regard to counseling patients on mid‐term postoperative expectations after AFFs.

## Disclosures

The authors have no conflicts of interest to declare. The manuscript, including all data, figures, tables, and supplementary materials, has not been previously reported or published and will not be submitted to another journal while under review by JBMR Plus. The data included has not been the subject of previous publications.

## Author Contributions

**Jonathon Spanyer:** Conceptualization; formal analysis; project administration; supervision; validation; writing‐original draft. **Lauren Barber:** Data curation; investigation; writing‐original draft; writing‐review & editing. **Harrison Lands:** Data curation; investigation; writing‐original draft; writing‐review & editing.**Alexander Brown:** Data curation; investigation; project administration; validation; writing‐review & editing. **Mary Bouxsein:** Conceptualization; formal analysis; project administration; supervision; validation; writing‐review & editing. **Marilyn Heng:** Conceptualization; formal analysis; project administration; supervision; writing‐original draft; writing‐review & editing. **Madhusudhan Yakkanti:** Conceptualization; investigation; methodology; project administration; supervision; writing‐review & editing.

### Peer Review

The peer review history for this article is available at https://publons.com/publon/10.1002/jbm4.10514.
